# Protective effects of *Bacillus* probiotics against high-fat diet-induced metabolic disorders in mice

**DOI:** 10.1371/journal.pone.0210120

**Published:** 2018-12-31

**Authors:** Bobae Kim, Jeonghyeon Kwon, Min-Seok Kim, Haryung Park, Yosep Ji, Wilhelm Holzapfel, Chang-Kee Hyun

**Affiliations:** 1 School of Life Science, Handong Global University, Pohang, Gyungbuk, Republic of Korea; 2 Department of Advanced Green Energy and Environment (AGEE), Handong Global University, Pohang, Gyungbuk, Republic of Korea; 3 Holzapfel Effective Microbes (HEM), Pohang, Gyungbuk, Republic of Korea; Universidade do Estado do Rio de Janeiro, BRAZIL

## Abstract

Recently, modulation of gut microbiota by probiotics treatment has been emerged as a promising strategy for treatment of metabolic disorders. Apart from lactic acid bacteria, *Bacillus* species (*Bacillus* spp.) have also been paid attention as potential probiotics, but nevertheless, the molecular mechanisms for their protective effect against metabolic dysfunction remain to be elucidated. In this study, we demonstrate that a probiotic mixture composed of 5 different *Bacillus* spp. protects mice from high-fat diet (HFD)-induced obesity, insulin resistance and non-alcoholic fatty liver disease (NAFLD). Probiotic *Bacillus* treatment substantially attenuated body weight gain and enhanced glucose tolerance by sensitizing insulin action in skeletal muscle and epididymal adipose tissue (EAT) of HFD-fed mice. *Bacillus*-treated HFD-fed mice also exhibited significantly suppressed chronic inflammation in the liver, EAT and skeletal muscle, which was observed to be associated with reduced HFD-induced intestinal permeability and enhanced adiponectin production. Additionally, *Bacillus* treatment significantly reversed HFD-induced hepatic steatosis. In *Bacillus*-treated mice, hepatic expression of lipid oxidative genes was significantly increased, and lipid accumulation in subcutaneous and mesenteric adipose tissues were significantly decreased, commensurate with down-regulated expression of genes involved in lipid uptake and lipogenesis. Although, in *Bacillus*-treated mice, significant alterations in gut microbiota composition was not observed, the enhanced expression of tight junction-associated proteins showed a possibility of improving gut barrier function by *Bacillus* treatment. Our findings provide possible explanations how *Bacillus* probiotics protect diet-induced obese mice against metabolic disorders, identifying the treatment of probiotic *Bacillus* as a potential therapeutic approach.

## Introduction

Overnutrition triggers excessive ectopic lipid accumulation, increases low-grade chronic inflammation and suppresses insulin signaling pathway, leading to the development of metabolic diseases such as obesity, insulin resistance, dyslipidemia and non-alcoholic fatty liver disease (NAFLD) [1]. Accumulating evidence indicate that the imbalanced gut microbiota, interacting with inflammation and lipid metabolic dysregulation, plays a key role in the development of metabolic disorders [2]. From this perspective, probiotic modulation of gut microbiota has gained their reputation as a novel approach for the prevention and treatment of immune-mediated metabolic diseases [3]. Although the most common types of probiotics available are lactic acid bacteria [4], many *Bacillus* species (*Bacillus* spp.) have also been used extensively in humans as dietary probiotic supplements, in animals as growth promoters and in aquaculture for enhancing growth and disease-resistance [5]. The members of genus *Bacillus*, bacterial endospore formers, are able to survive the harshly acidic pH of stomach and can reach the small intestine to exert their probiotic properties. Probiotics *Bacillus* species being used include *B*. *subtilis*, *B*. *cereus*, *B*. *licheniformis*, *B*. *pumilus*, *B*. *clausii*, *B*. *coagulans*, *B*. *sonorensis* [6,7]. Several studies have demonstrated that *Bacillus* species have various probiotic activities including maintenance of intestinal homeostasis, competitive exclusion of pathogens and modulation of host immune system [8]. Probiotic *Bacillus* strains have also been shown to possess the ability of ameliorating gut microbiota dysbiosis and inflammation [6].

Recently, studies on the probiotic effect of *Bacillus* species have been extended to the treatment of metabolic disorders [6]. It was reported that dietary supplementation with *B*. *licheniformis*-fermented soybean paste prevented weight gain and improved glucose tolerance in high-fat diet (HFD)-induced obese mice [9]. Lee et al. [10] have demonstrated that a combination of soya pulp and *B*. *coagulans* improved bile acid metabolic dysfunction and NAFLD in rats fed a cholic acid-supplemented diet. An exopolysaccharide purified from *B*. *subtilis* was also shown to reduce serum glucose and cholesterol levels in streptozotocin-induced diabetic rats [11]. However, although several physiological observations on *Bacillus* probiotic improvement of metabolic dysfunctions have been reported through these studies, the mechanism underlying the effect has not been suggested yet.

We have previously reported that treatment of diet-induced obese (DIO) mice with a long-term fermented soybean paste (LFSP) improves non-alcoholic fatty liver disease (NAFLD) and insulin resistance, which was found to be exerted by its high bacterial content, especially *Bacillus* strains [12]. In this study, to examine how the *Bacillus* probiotics exert protective effects against metabolic disorders, mice were treated with a probiotic supplement composed of 5 *Bacillus* strains isolated from LFSP. Our data showed that *Bacillus* treatment attenuated weight gain, glucose intolerance and NAFLD, which was associated with suppression of chronic inflammation, beneficial alteration in lipid metabolism, and enhancement of gut barrier function. These findings suggest that *Bacillus*-based probiotic formulation has a big potential to be utilized as clinically relevant therapeutics for the treatment of metabolic disorders.

## Materials and methods

### Bacterial strains and culture conditions

*B*. *sonorensis* JJY12-3, *B*. *paralicheniformis* JJY12-8, *B*. *sonorensis* JJY13-1, *B*. *sonorensis* JJY 13–3, and *B*. *sonorensis* JJY 13–8 were isolated from long-term fermented soybean paste [12]. Each strain has been deposited in Korean Collection for Type Cultures (KCTC; WDCM597) under the number KCTC 13405BP, 13406BP, 13407BP, 13408BP, and 13409BP. The strains were grown in BD Difco LB Broth (Becton, Dickinson and Company, Franklin Lakes, NJ) at 37 °C. For administration to mice, a bacterial cell mixture of the 5 *Bacillus* strains was daily prepared by mixing each of the strains with an equal cell count to get a total concentration of 1 x 10^8^ CFU/200 μL PBS. To compare the probiotic activity of the *Bacillus* mixture, VSL#3, a formulation of 4 strains of *Lactobacillus*, 3 strains of *Bifidobacteria* and 1 strain of *Streptococcus thermophilus*, was used as a positive control at a concentration of 1 x 10^8^ CFU/200 μL PBS [13].

### Animal experiments

Five-week-old C57BL/6J male mice supplied by Hyochang Bioscience (Daegu, Korea) were maintained in humidity and temperature-controlled environment (22 ± 1 °C and 45 ± 10%) on a 12 h light/dark cycle. After 1 week of acclimatization with ND feeding (2018S, Harlan Laboratories, Indianapolis, IN, USA), mice were divided into four groups (n = 9–10 per groups); normal diet (ND)-fed control, HFD-fed control, HFD-fed VSL#3-treated, and HFD-fed *Bacillus*-treated groups. Each group was fed with ND or HFD (60%kcal from fat, D12492, Research Diets Inc., New Brunswick, NJ, USA) for 2 weeks, and then during following 13 weeks of ND or HFD feeding, mice received gavage with 200 μL PBS or a daily dose of 1 x 10^8^ CFU (suspended in 200 μL PBS, once a day) VSL#3 or *Bacillus*. At the end of 13-week treatment, mice were starved for 4 h and sacrificed by cervical dislocation under ether anesthesia. For examining insulin-stimulated Akt phosphorylation, mice of each group were injected with insulin (n = 4) or PBS (n = 5~6) for 10 min before sacrifice. To collect serum sample, 500 μl of blood samples were drawn from heart, coagulated for 1 h, and centrifuged at 2,000 g for 30 min. Tissues of the liver, quadriceps skeletal muscle, subcutaneous adipose tissue (SAT), epididymal adipose tissue (EAT), mesenteric adipose tissue (MAT), and interscapular brown adipose tissue (BAT) were harvested, snap-frozen in liquid nitrogen, and stored at -70 °C until processed for RNA and protein analysis. For fecal bacteria analysis, the bedding for the mice was changed 1 day before sacrifice, and all the stools collected from each bedding were put in one vial and stored at -70 °C until bacterial DNA extraction from whole fecal sample. All animal experiments were performed in accordance with protocols approved by the Committee on the Ethics of Animal Experiments of the Handong Global University (Permit number: 20160616–008).

### Serum analyses

Serum insulin level was assayed by sandwich ELISA method using Ultra Sensitive Mouse Insulin ELISA kit (Morinaga Institute of biological Science, Yokohama, Japan). Serum lipopolysaccharide (LPS) level was measured using ToxinSensor Chromogenic LAL Endotoxin Assay kit (GenScript, Piscataway, NJ, USA).

### Glucose tolerance test

After 15-week of VSL#3 or *Bacillus* treatment, mice were fasted for 16 h, with free access to water, prior to the test. Glucose was injected intraperitoneally at a concentration of 2 g/kg body weight, followed by collection of tail blood samples and measurement of blood glucose levels at 0, 15, 30, 60, 90, and 120 min after glucose injection by GlucoDr auto AGM-4000 (Allmedicus, Anyang, Korea).

### Histological analysis

Liver, MAT and SAT samples fixed in 10% v/v formalin/PBS were embedded in paraffin, and then 5-μm-thick microtome sections were prepared and stained with hematoxylin and eosin. Images were obtained under a microscope at a magnification of 200x and the areas of adipocytes were measured using the ImageJ software with Adiposoft plug-in according to the developer’s instruction [14].

### Liver TG quantification

Liver sample (75 mg) was mechanically homogenized for 1 min with a hand-held disperser (IKA T10, Staufen, Germany) in 1.5 ml of chloroform/methanol (2:1) solution, and then shaking incubated for 2–3 h at room temperature. After adding 150 μL of 1 M H_2_SO_4_, the whole-lysate was centrifuged at 2,000 rpm for 20 min. The bottom layer (750 μL) containing triglycerides (TG) and phospholipids was transferred to new vial, to which 750 μL of 1% Triton X-100/chloroform was added, and then dried overnight at room temperature. Dried sample was reconstituted with water, and TG level was measured with TG-S assay kit (Asan Pharm. Co. Ltd, Seoul, Korea) according to the instructions of the manufacturer.

### Gut microbiota analysis and short chain fatty acid (SCFA) quantification

For gut microbiota analysis, microbial genomic DNA extraction from fecal sample was performed as described by Ji et al. [15]. The microbiota composition of fecal sample was characterized by real-time PCR based Gut Low-Density Array (GULDA) according to the method of Bergström et al. [16]. The targeted bacterial groups, including *Firmicutes*, *Bacteroidetes*, *Bacteroides spp*., and *Prevotella spp*., were detected with the group-specific probes. The relative abundance of each bacterial gene target was determined by normalizing with a universal bacterial primer-target. For the analysis of SCFA in blood samples, the amount of SCFAs including acetate, propionate, and butyrate in serum was measured using Shimadzu GC-2010 gas chromatography system (Shimadzu Scientific Instruments, Kyoto, Japan) as described by Ji et al. [15].

### Real-time RT PCR

Total RNA extraction, reverse transcription and quantitative PCR were conducted as described previously [17]. Briefly, total RNA was extracted with TRI reagent (Molecular Research, Cincinnati, OH, USA) and was transcribed to complementary DNA using GoScript Reverse Transcriptase (Promega, Madison, WI, USA) following the instructions of the manufacturer. Quantitative real-time PCR was performed by SYBR Premix Ex Taq II (Takara Bio Inc., Shiga, Japan) and gene-specific forward and reverse primers on an ABI 7500 Fast Real-Time PCR system (Applied Biosystems, Foster City, CA, USA). Quantification of gene transcripts for acetyl-CoA carboxylase (ACC), acyl-CoA oxidase 1 (Acox1), CD36, carnitine palmitoyltransferase 1 (CPT1), fatty acid synthase (FAS), interferon γ (INFγ), interleukin-1β (IL-1β), IL-6, IL-12, low-density lipoprotein receptor (LDLR), monocyte chemoattractant protein (MCP-1), occludin, peroxisome proliferator-activated receptor γ (PPARγ), stearoyl-CoA desaturase-1 (SCD1), sterol regulatory element-binding protein 1 (SREBP1c), tumor necrosis factor α (TNFα), zonula occludens-1 (ZO-1) was performed using gene-specific primers. Primers were designed using Primer-BLAST tool of National Center for Biotechnology Information (https://www.ncbi.nlm.nih.gov/tools/primer-blast/) and validated by SYBR green real-time PCR and agarose gel electrophoresis of the PCR products. Primer sequences are available in S2 Table. Thermocycling conditions for the PCR amplification were: 30 sec at 95°C, then 45 cycles of 5 sec at 95°C, and 30 sec at 60°C. Immediately after amplification, melting curve analysis was performed with a procedure consisting of heating up to and incubating at 95°C for 5 sec, cooling to 60 °C for 30 sec, and heating up at a ramp rate of 0.3°C/sec. Results were presented as means ± S.D. normalized to expression of acidic ribosomal phosphoprotein (Arbp) using the ΔΔ Ct method, in which the HF+PBS group was used as the reference group.

### Western blot analysis

Western blot analysis was carried out as described previously [17]. Antibodies against total Akt, phospho (Ser473) Akt, total AMPK, phospho (Thr172) AMPK, adiponectin, GAPDH (Cell signaling technology, Beverly, MA), occludin (Bioss antibodies, Woburn, MA), PGC1α (Santa Cruz Biotechnology, Santa Cruz, CA) were used as primary antibodies, with HRP-conjugated anti-rabbit IgG as secondary antibody (Cell signaling technology). Detailed information on the antibodies is available in S3 Table. Immunoblots were visualized by ECL, and densitometric analyses were done using ImageJ software.

### Statistical analyses

The experimental results were presented as means ± S.D for 6–8 mice in each group. Statistical analyses were performed using GraphPad Prism version 6 (GraphPad, La Jolla, CA, USA). Statistical comparisons were carried out using ordinary or repeated measure one-way analysis of variance (ANOVA) as indicated in each separate experiment with Tukey’s multiple comparison test with α = 0.05. *P* values < 0.05 were considered as statistically significant.

## Results

### *Bacillus* treatment protects mice against HFD-induced weight gain and insulin resistance

**Fig 1:**
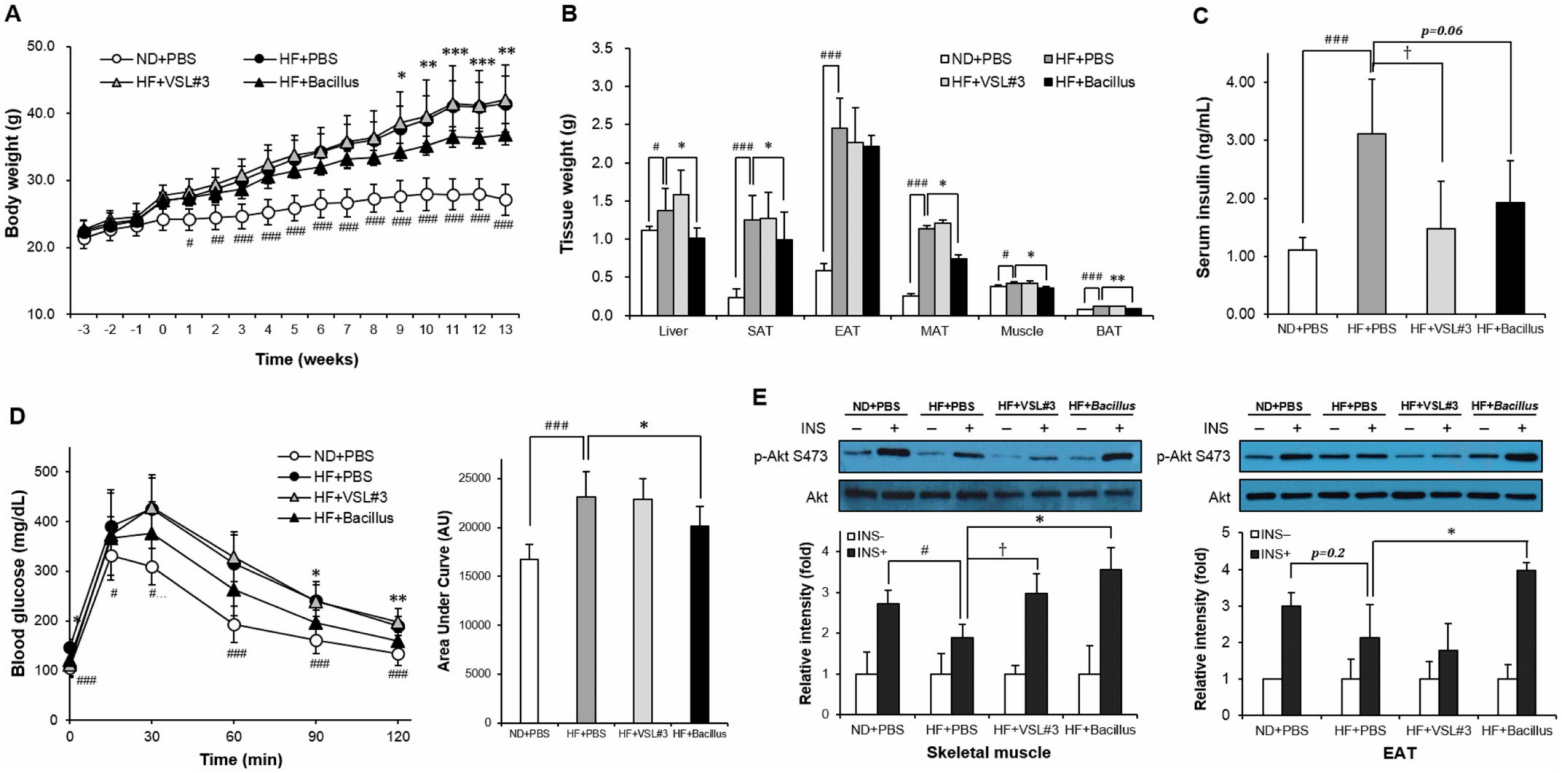
*Bacillus* treatment protects mice against excessive weight gain and insulin resistance. (A) Changes of body weight for 15 weeks of HF feeding with *Bacillus* treatment for latter 13 weeks (n = 9~10). (B) Changes of tissue weight after 13 weeks of *Bacillus* treatment (n = 9~10). (C) Serum concentration of insulin quantified by ELISA (n = 6). Serum sample was diluted 20-fold with dilution buffer and analyzed according to the manufacturer’s protocol. (D) Glucose tolerance test after 13 weeks of *Bacillus* treatment and the area under the curve (n = 8~10). The blood glucose levels were measured at 0, 15, 30, 60, 90 and 120 min after intraperitoneal injection of glucose (2 g/kg). (E) Effect of *Bacillus* treatment on insulin-stimulated Akt phosphorylation in skeletal muscle and EAT (n = 3). After 4 h fasting and intraperitoneal injection of insulin (0.75 U/kg) for 10 min, mice were sacrificed, and tissues were rapidly excised. Proteins were extracted from tissues for SDS-PAGE-immunoblot analysis. Data present mean ± SD of fold changes in blot intensity between PBS- and insulin-challenged subgroups in each experimental group. Differences between experimental groups were analyzed using repeated measure (Fig 1A and 1D GTT) or ordinary one-way ANOVA with Tukey’s multiple comparison test. # *p* < 0.05, ## *p* < 0.01, ### *p* < 0.001 between ND+PBS and HF+PBS, † *p* < 0.05 between HF+PBS and HF+VSL#3, * *p* < 0.05, ** *p* < 0.01, *** *p* < 0.001 between HF+PBS and HF+*Bacillus*. ND: normal chow diet, HF: high-fat diet, PBS: phosphate buffered saline, INS: insulin, SAT: subcutaneous adipose tissue, EAT: epididymal adipose tissue, MAT: mesenteric adipose tissue, BAT: interscapular adipose tissue.

Mice on a HFD treated with *Bacillus* had significantly lower body weight gain when compared to their control (HF+PBS) mice (*p* < 0.05) (Fig 1A), while VSL#3-treated (HF+VSL#3) mice did not show any change in weight gain. The suppression of HFD-induced weight gain in *Bacillus*-treated mice was in parallel with significant reduction in the weights of tissues including SAT (*p* = 0.04), MAT (*p* = 0.02), BAT (*p* = 0.002), skeletal muscle (*p* = 0.03), and the liver (*p* = 0.01) (Fig 1B). Blood glucose concentration was reduced at 0, 90, and 120 min (*p* < 0.05) with no significant change of serum insulin levels (*p* = 0.07) in *Bacillus*-treated, but not VSL#3-treated, mice compared to HFD-fed control mice (Fig 1C and 1D). It was also observed that the insulin-stimulated Akt phosphorylation at Ser473 was significantly increased in skeletal muscle (*p* = 0.01) and EAT (*p* = 0.04) of *Bacillus*-treated mice (Fig 1E) compared to that of HFD-fed control mice, showing the improved insulin signaling. Together, these results indicate that *Bacillus* improves glucose tolerance with is associated with enhanced insulin sensitivity.

### *Bacillus* treatment suppresses HFD-induced chronic inflammation in the liver, EAT and skeletal muscle

**Fig 2:**
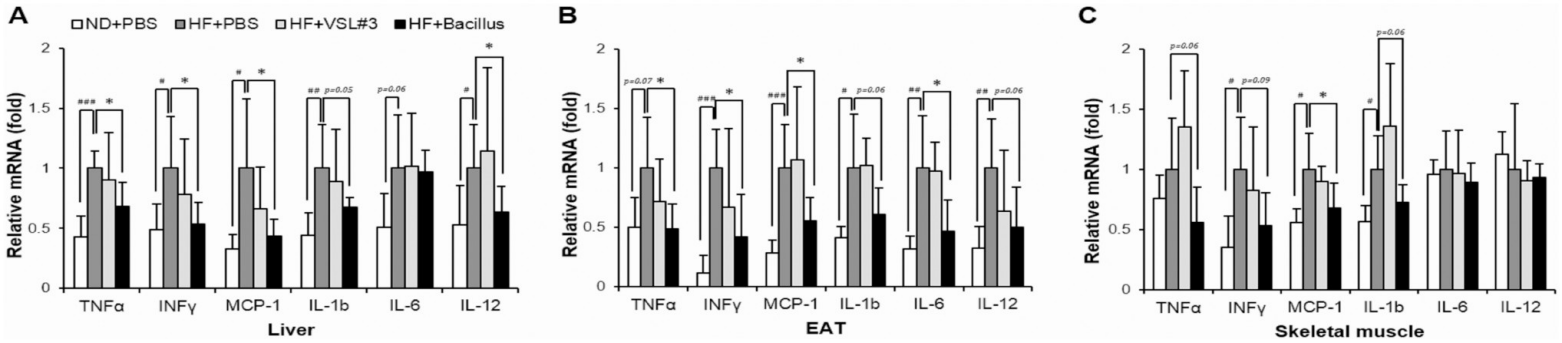
*Bacillus* treatment suppresses chronic inflammation in the liver, EAT and skeletal muscle. Effect of Bacillus treatment on mRNA expression levels of pro-inflammatory cytokines in (A) the liver, (B) EAT and (C) skeletal muscle. Total RNA extracted from tissues were reverse transcribed, and each gene expression was quantified by real-time PCR using gene-specific primers. All genes are normalized to expression of Arbp. Data present mean ± SD for 5~6 mice in each group. Differences between experimental groups were analyzed using one-way ANOVA with Tukey’s multiple comparison test. # *p* < 0.05, ## *p* < 0.01, ### *p* < 0.001 between ND+PBS and HF+PBS, * *p* < 0.05 between HF+PBS and HF+*Bacillus*. ND: normal chow diet, HF: high-fat diet, PBS: phosphate buffered saline, EAT: epididymal adipose tissue.

Chronic inflammation is a major cause of obesity-induced insulin resistance [18]. To examine whether *Bacillus* treatment improved chronic inflammation in HFD-fed mice, we analyzed mRNA expression of pro-inflammatory cytokines in the liver, EAT, and skeletal muscle. In the liver of *Bacillus*-treated mice, mRNA expression of TNFα (*p* = 0.01), INFγ (*p* = 0.04), MCP-1 (*p* = 0.03), and IL-12 (*p* = 0.04) were significantly decreased, and IL-1β expression was moderately decreased without reaching statistical significance (*p* = 0.05) (Fig 2A). Similarly, in EAT of *Bacillus*-treated mice, mRNA expression of TNFα (*p* = 0.02), INFγ (*p* = 0.03), MCP-1 (*p* = 0.02), and IL-6 (*p* = 0.04) were significantly decreased, and expression of IL-1β and IL-12 were moderately decreased without statistical significance (*p* = 0.06) (Fig 2B). Additionally, skeletal muscle of *Bacillus*-treated mice also had significantly lower mRNA level of MCP-1 (*p* = 0.04), and levels of TNFα, IL-1β (*p* = 0.06), and INFγ (*p* = 0.09) tended to be lower, but without significance (Fig 2C). These reductions in the expression of pro-inflammatory cytokines were not observed in VSL#3-treated mice (Fig 2). These results together suggested that *Bacillus* treatment might suppress HFD-induced pro-inflammatory response, which contributed to the enhancement of insulin sensitivity.

### *Bacillus* treatment enhances adiponectin production and reduces intestinal permeability

**Fig 3:**
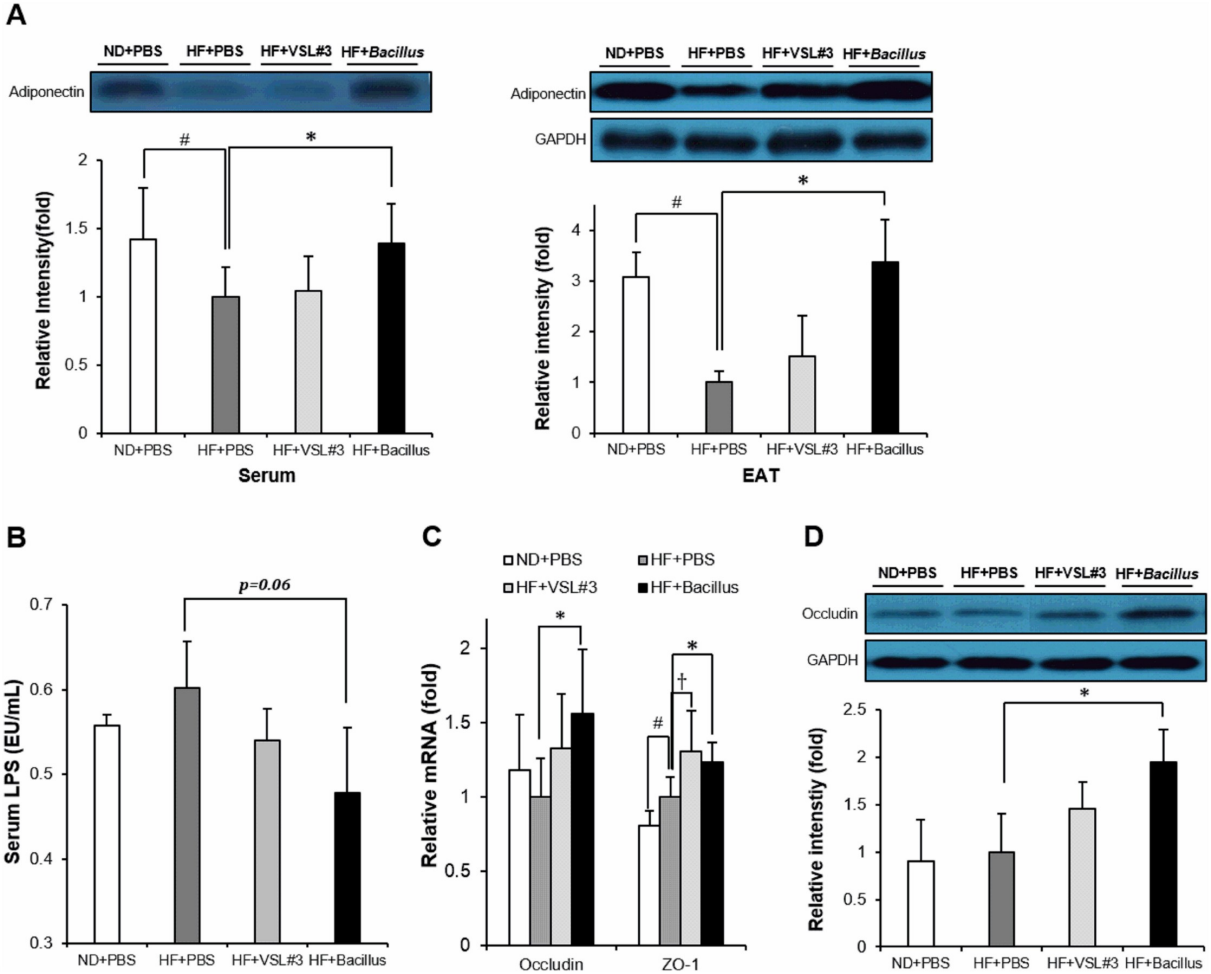
*Bacillus* treatment reverses HFD-induced deterioration in adiponectin production and enhances intestinal barrier function. (A) Effect of *Bacillus* treatment on adiponectin levels in serum and EAT (n = 4~5). (B) Serum concentration of LPS quantified by chromogenic LAL endotoxin assay kit according to the manufacturer’s protocol (n = 3~5). (C) Effect of *Bacillus* treatment on mRNA levels associated with intestinal permeability in ileum (n = 5~6). All genes are normalized to expression of Arbp. (D) Effect of *Bacillus* treatment on Occludin protein levels in ileum (n = 4~5). Proteins were extracted from ileum for SDS-PAGE-immunoblot analysis. GAPDH was used as a loading control. Data present mean ± SD of fold changes in blot intensity between experimental groups. Differences between experimental groups were analyzed using one-way ANOVA with Tukey’s multiple comparison test. # *p* < 0.05 between ND+PBS and HF+PBS, † *p* < 0.05 between HF+PBS and HF+VSL#3, * *p* < 0.05 between HF+PBS and HF+*Bacillus*. ND: normal chow diet, HF: high-fat diet, PBS: phosphate buffered saline, EAT: epididymal adipose tissue, LPS: lipopolysaccharide.

To examine how *Bacillus* treatment improved HFD-induced chronic inflammation, we assessed adiponectin production and intestinal permeability modulation. Adiponectin is an anti-inflammatory adipokine that modulates metabolic dysfunction in obesity and protects against metabolic disorders [19]. Exacerbation of intestinal permeability leading to increased circulating LPS level is also an important factor aggravating chronic inflammation under HFD-fed condition [20]. In the present study, adiponectin levels in serum (*p* = 0.03) and EAT (*p* = 0.01) were significantly higher in *Bacillus*-treated, but not VSL#3-treated, mice than those in HFD-fed control mice (Fig 3A). *Bacillus*-treated mice also showed a significant increase in mRNA expression of tight junction-associated proteins, occludin (*p* = 0.04) and ZO-1 (*p* = 0.02), in ileum (Fig 3C). Consistent with mRNA expression levels, occludin protein level was also observed to be significantly higher in *Bacillus*-treated mice (*p* = 0.04) (Fig 3D). Unexpectedly, however, the serum LPS level was not significantly lower in *Bacillus*-treated mice than that of HFD-fed control mice (*p* = 0.06) (Fig 3B). VSL#3 treatment, however, exhibited no change in serum LPS level and the expression of occludin (Fig 3B–3D). Together, these results showed that protective effect of *Bacillus* treatment against chronic inflammation was associated, at least in part, with enhanced adiponectin production and intestinal barrier function.

### *Bacillus* treatment lowers fat accumulation and increases fatty acid oxidation in the liver

**Fig 4:**
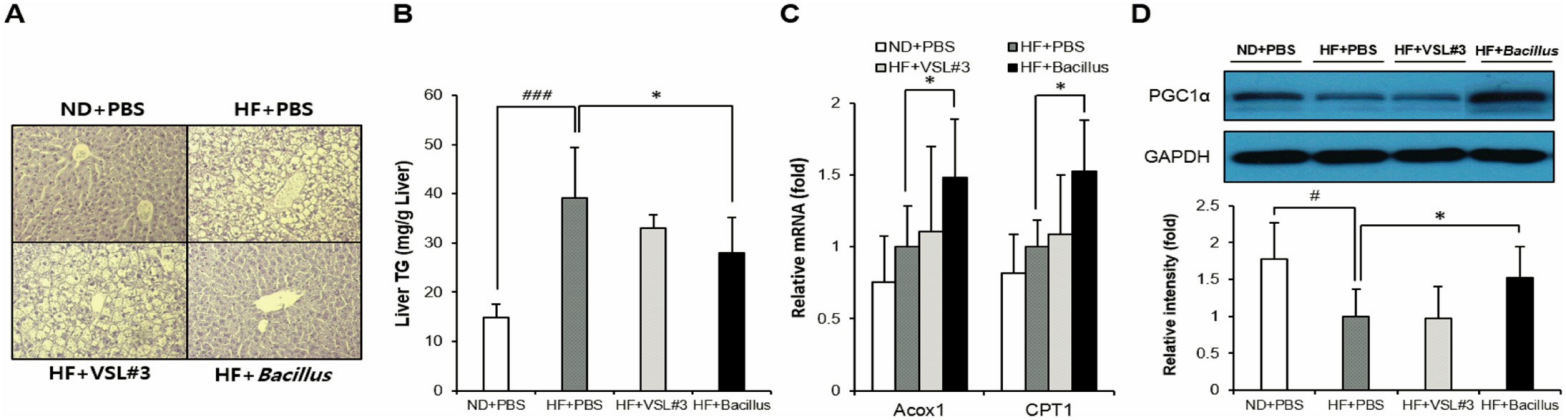
*Bacillus* treatment improves hepatic steatosis and increases fatty acid oxidation. (A) Changes of hepatic adiposity after 13 weeks of *Bacillus* treatment. Three representative mice of each group were selected to compare histological features between groups. Shown are representative photomicrographs of liver sections stained with hematoxylin and eosin (200X). (B) Effect of *Bacillus* treatment on the liver TG accumulation (n = 9~10). Hepatic lipids were extracted by homogenizing the liver tissue in chloroform/methanol lipid extraction buffer and analyzed according to the manufacturer’s protocol. (C) Changes of lipid oxidative gene expressions in the liver (n = 5~6). All genes are normalized to expression of Arbp. (D) Effect of *Bacillus* treatment on hepatic PGC1α protein level (n = 4~5). Proteins were extracted from the liver and analyzed by SDS-PAGE-immunoblotting. GAPDH was used as a loading control. Data present mean ± SD of fold changes in blot intensity between experimental groups. Differences between experimental groups were analyzed using one-way ANOVA with Tukey’s multiple comparison test. # *p* < 0.05, ### *p* < 0.001 between ND+PBS and HF+PBS, * *p* < 0.05 between HF+PBS and HF+*Bacillus*. ND: normal chow diet, HF: high-fat diet, PBS: phosphate buffered saline, TG: triacylglycerol.

Next, to test whether the significant attenuation in hepatic inflammation in *Bacillus*-treated mice was accompanied with an improvement of hepatic steatosis, histological examination was performed on the liver. *Bacillus*-treated, but not VSL#3-treated, mice exhibited a significantly reduced fat deposition in hepatocytes compared with HFD-fed control mice (Fig 4A). Consistent with this histological observation, the hepatic triglyceride (TG) accumulation appeared to be significantly lower in *Bacillus*-treated (*p* = 0.01), but not VSL#3-treated, mice than their HFD-fed controls (Fig 4B). When the hepatic mRNA expression of lipogenic genes was measured, no significant difference between *Bacillus*-treated mice and their HFD-fed controls was observed (S1 Fig). However, the analysis of hepatic mRNA expression of genes related to fatty acid oxidation showed significant increases in the expressions of Acox1 (*p* = 0.04) and CPT1 (*p* = 0.02) in *Bacillus*-treated, but not VSL#3-treated, mice compared to their HFD-fed controls (Fig 4C). In addition, the level of hepatic PGC1α protein was higher in *Bacillus*-treated, but not VSL#3-treated, mice than their HFD-fed controls (*p* = 0.04) (Fig 4D). Therefore, these data demonstrated that *Bacillus* treatment had a protective effect against hepatic steatosis through increasing fatty acid oxidation without altering lipogenesis, which was not exerted by VSL#3 treatment.

### *Bacillus* treatment suppresses fat accumulation in SAT and MAT

**Fig 5:**
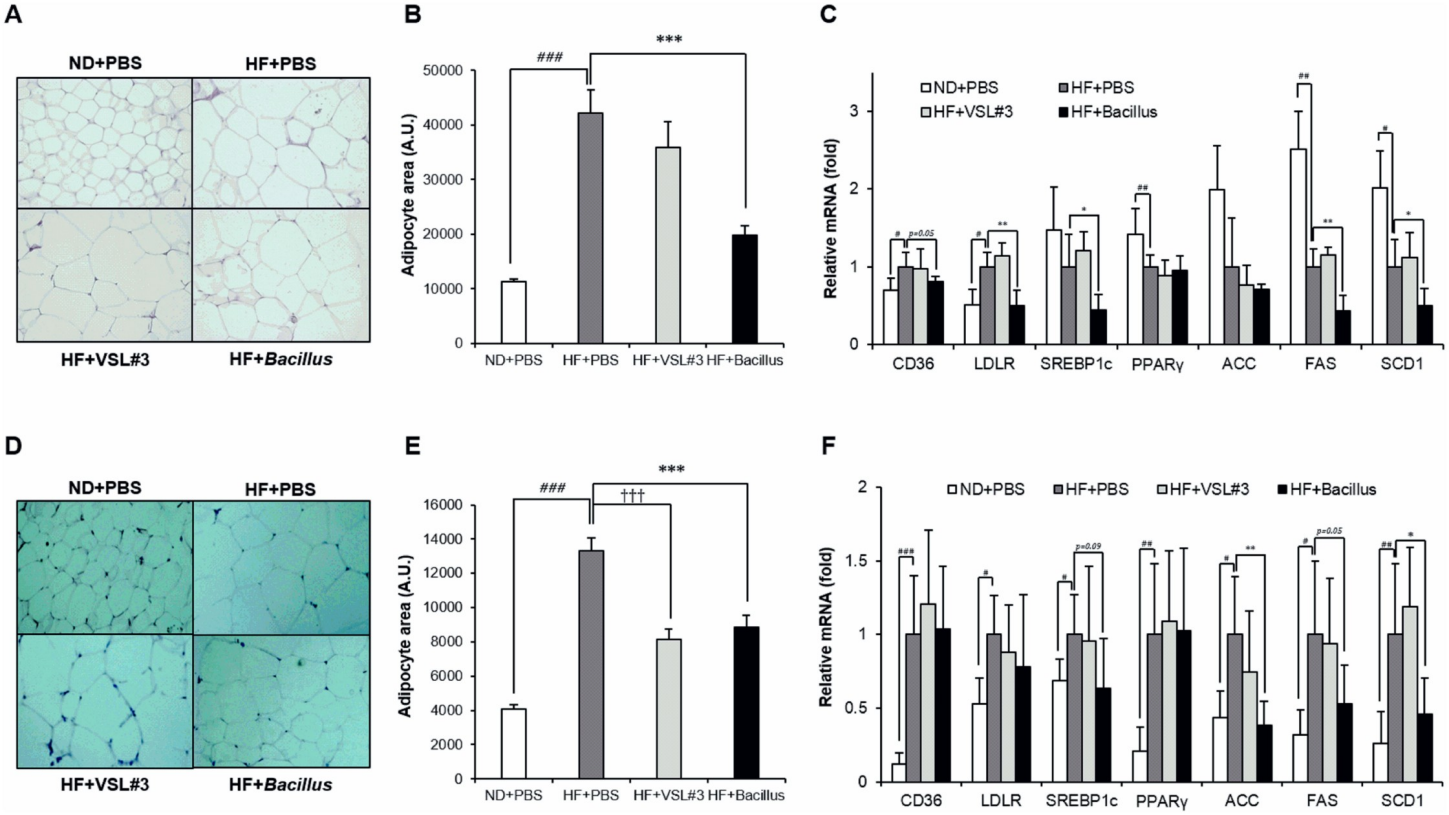
*Bacillus* treatment suppresses lipid accumulation in subcutaneous and mesenteric adipose tissues. (A and B) Changes in adipocyte size in SAT after 13 weeks of *Bacillus* mixture treatment (n = 3). Three representative mice of each group were selected to compare histological features between groups. Shown are representative photomicrographs of SAT sections stained with hematoxylin and eosin (200X). (C) Effect of *Bacillus* treatment on mRNA expression levels of lipid uptake and synthesis in SAT (n = 5~6). (D and E) Changes in adipocyte size in MAT (n = 3) with representative photomicrographs. (F) mRNA expression levels related to lipid uptake and synthesis in MAT (n = 5~6). All genes are normalized to expression of Arbp. Data present mean ± SD. Differences between experimental groups were analyzed using one-way ANOVA with Tukey’s multiple comparison test. # *p* < 0.05, ## *p* < 0.01, ### *p* < 0.001 between ND+PBS and HF+PBS, ††† *p* < 0.001 between HF+PBS and HF+VSL#3, * *p* < 0.05, ** *p* < 0.01, *** *p* < 0.001 between HF+PBS and HF+*Bacillus*. ND: normal chow diet, HF: high-fat diet, PBS: phosphate buffered saline, SAT: subcutaneous adipose tissue, MAT: mesenteric adipose tissue.

To better understand how *Bacillus* treatment reduced adiposity of SAT and MAT (Fig 1B), we analyzed lipid metabolism in adipose tissues. The size of adipocytes was significantly lower both in SAT and MAT of *Bacillus*-treated mice than that of their HFD-fed controls (*p* < 0.001) (Fig 5A, 5B, 5D and 5E). This decrease in adipocyte size was corroborated by reduced expression of genes involved in lipid uptake and lipogenesis both in SAT and MAT (Fig 5C and 5F). The expression level of lipid uptake genes such as CD36 (SAT *p* = 0.05) and LDLR (SAT *p* = 0.003) and lipogenic genes such as SREBP1c (SAT *p* = 0.03, EAT *p* = 0.09), ACC (EAT *p* = 0.01), FAS (SAT *p* = 0.01, EAT *p* = 0.05), and SCD1 (SAT *p* = 0.04, EAT *p* = 0.04) was lower in *Bacillus*-treated mice than that of their HFD-fed controls. However, the expression of lipid oxidative genes was not altered by *Bacillus* treatment (S2 Fig).

### *Bacillus* treatment might not be directly associated with HFD-induced gut microbiota dysbiosis

To examine whether the beneficial metabolic effects of *Bacillus* treatment is associated with an amelioration of HFD-induced microbiota alterations, gut microbiota compositions in feces were analyzed and compared. It has been reported that HFD-fed mice showed increased Firmicutes and decreased Bacteroidetes [21], and also had an increased proportion of *Bacteroides* and a reduction in *Prevotella* [22]. In this study, *Bacillus*-treated mice showed some changes in Firmicutes/Bacteroidetes (*p* = 0.11) and *Bacteroides*/*Prevotella* (*p* = 0.18) ratios compared to control mice (S3A Fig), however, there was no statistical difference. We next analyzed serum levels of SCFAs to corroborate the microbiota-modulating effect of *Bacillus* treatment. Among SCFAs, acetate is known to be a kind of short chain fatty acids produced by microbial fermentative activity in the gut, and also the principal product of *Bacteroides* spp. [23,24]. We observed that the level of serum acetate was significantly lower in *Bacillus*-treated mice than that of their controls (*p* = 0.002) (S3B Fig), which was correlated with reduced abundance of *Bacteroides*. Consistent with this, the expression of GPR43, the acetate receptor, in the liver (*p* = 0.01) and EAT (*p* = 0.04) was decreased in *Bacillus*-treated mice compared to their controls (S3C Fig).

### Each *Bacillus* strain exerts strain-specific protective effect against HFD-induced body weight gain, glucose intolerance and hepatic steatosis

**Fig 6:**
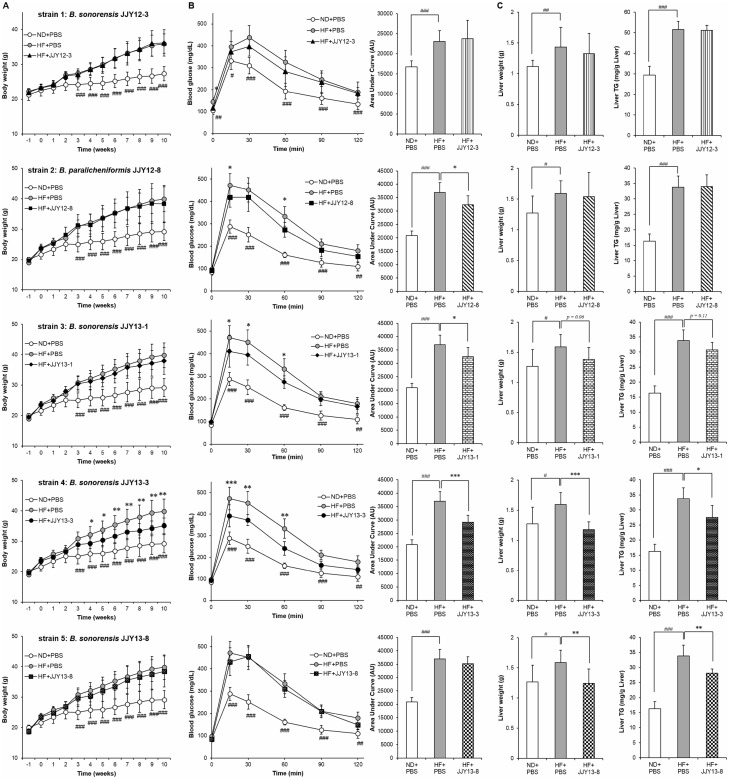
*S*train-dependent effects of *Bacillus* strains on improvements of metabolic dysfunctions. Mice on a HFD were treated with each individual *Bacillus* strain for 10 weeks. Changes of (A) body weight (n = 8), (B) glucose tolerance test, and (C) hepatic TG level (n = 6~8). Data present mean ± SD. Differences between experimental groups were analyzed using repeated measure (Fig 6A and 6B GTT) or ordinary one-way ANOVA with Tukey’s multiple comparison test. # *p* < 0.05, ## *p* < 0.01, ### *p* < 0.001 between ND+PBS and HF+PBS, * *p* < 0.05, ** *p* < 0.01, *** *p* < 0.001 between HF+PBS and HF+*Bacillus* strains. ND: normal chow diet, HF: high-fat diet, PBS: phosphate buffered saline.

Next, the beneficial effects on glucose and lipid metabolism observed in mice treated with *Bacillus* mixture shown above led us to examine the effect of each individual *Bacillus* strain constituting the *Bacillus* mixture. When daily treated with each individual strain for 10 weeks, mice exhibited strain-specific amelioration in HFD-induced body weight gain, glucose intolerance and hepatic fat deposition (Fig 6). Among five *Bacillus* strains, mice treated with *B*. *sonorensis* JJY13-3 showed a reduction in body weight gain compared to HFD-fed control mice (*p* < 0.05), whereas there were no changes in mice treated with each of the other four strains (Fig 6A). Mice treated with *B*. *paralichemiformis* JJY12-8 (AUC *p* = 0.02), *B*. *sonorensis* JJY13-1 (AUC *p* = 0.02) or *B*. *sonorensis* JJY13-3 (AUC *p* < 0.001) had improved glucose tolerance compared to their HFD-fed controls (Fig 6B). It was also observed that HFD-induced hepatomegaly and hepatic TG accumulation were alleviated by treatment of *B*. *sonorensis* JJY13-3 (liver weight *p* < 0.001, liver TG *p* = 0.01) or *B*. *sonorensis* JJY13-8 (liver weight *p* = 0.01, liver TG *p* = 0.005) (Fig 6C). From these results, it was suggested that the protective effect of *Bacillus* mixture against metabolic dysfunctions was a combination of these strain-specific effects.

## Discussion

It has been proposed that manipulation of gut microbiota with probiotics could be a potential therapeutic strategy for metabolic disorders such as obesity, insulin resistance and NAFLD [25]. Aside from lactic acid bacteria, of which value has been well acknowledged both scientifically and commercially [26], *Bacillus* spp. have gained attention as potential probiotic strains and have been commercialized in the form of diverse range of health supplements [6]. Although recent studies have proposed improving effects of *Bacillus*-containing supplements on metabolic dysfunctions [9–11], the mechanisms underlying the improvements still remain to be elucidated. We previously reported the protective effect of Korean traditional fermented soybean paste, which has high diversity and richness of *Bacillus* probiotic strains, against HFD-induced insulin resistance and NAFLD [12]. In the present study, we found that *Bacillus* strains isolated from the fermented soybean paste had protective effects against HFD-induced metabolic impairments, and investigated the molecular mechanisms underlying the effects.

The treatment of a probiotic mixture containing 5 *Bacillus* strains attenuated HFD-induced body weight gain, which was attributed to reduced fat accumulation in SAT and MAT (Figs 1A, 1B and 5). In parallel with a reduction in body weight gain, *Bacillus* treatment improved insulin sensitivity in skeletal muscle and EAT, confirmed by enhanced insulin-stimulated Akt phosphorylation (Fig 1C–1E).

Obesity-associated chronic inflammation is known to be responsible for the impaired insulin sensitivity [2]. In our study, it was observed that *Bacillus* treatment downregulated the expression of pro-inflammatory cytokines including TNFα, IFNγ, MCP1, IL-1β, IL-6 and IL-12 in the liver, EAT and skeletal muscle of HFD-fed mice (Fig 2). This observation implies that the enhancement of insulin sensitivity by *Bacillus* treatment was ascribed to the suppression of HFD-induced chronic inflammation in the peripheral tissues. We also found that *Bacillus* treatment significantly restored the impaired production of adiponectin under HFD-fed condition (Fig 3A). Adiponectin, exclusively produced from adipose tissues, is known to improve insulin sensitivity and attenuate chronic inflammation in peripheral tissues [19]. These data together suggest that the adiponectin-mediated suppression of chronic inflammation contributes, at least in part, to the improved insulin resistance in *Bacillus*-treated mice.

It has been reported that a prolonged high-fat feeding alters intestinal barrier structure via a reduced expression of tight junction proteins, followed by elevation of circulating endotoxin, i.e. LPS, consequently contributing to the onset of chronic inflammation in peripheral tissues [20,27]. It also has been demonstrated that enhanced intestinal barrier function improves insulin sensitivity through reduction of LPS leakage [28]. In our study, even though the reduction in serum LPS level did not show statistical significance (Fig 3B), the expression of tight junction-associated proteins, ZO-1 and occludin, was significantly increased in *Bacillus*-treated mice (Fig 3C and 3D), suggesting that *Bacillus* supplementation may contribute to the suppression of intestinal permeability.

Chronic liver diseases such as NAFLD and non-alcoholic steatohepatitis (NASH) are conditions of excessive fat accumulation and chronic inflammation in the liver [29]. Recently, it has become evident that, based on the close link between gut microbiota dysbiosis and chronic liver diseases, probiotics ameliorate NAFLD and NASH through manipulating the gut microbiota composition [3,30]. In our study, *Bacillus* treatment significantly suppressed HFD-induced hepatomegaly and hepatic steatosis (Figs 1B, 4A and 4B), which was corroborated by upregulated hepatic expression of lipid oxidative genes (Fig 4C and 4D). These data indicate that the *Bacillus* treatment-induced enhancement of hepatic fat oxidation, which leads to a reduction in fat accumulation and chronic inflammation, contributes to its protective effect against chronic liver diseases.

Adipocyte hypertrophy is a condition of excessive lipid accumulation beyond the adipocyte buffering capacity, which correlates positively with metabolic disorders [31,32]. Hypertrophy of adipocytes ensues various metabolic dysregulations such as tissue hypoxia, endoplasmic reticulum and oxidative stress, chronic inflammation, and ectopic fat deposition [33,34]. In this study, in addition to the significant reduction of hepatic adiposity, *Bacillus* treatment also exerted a suppressing effect on the adiposity of SAT and MAT (Fig 1B), which was commensurate with significantly reduced adipocyte hypertrophy in SAT and MAT of *Bacillus*-treated mice (Fig 5A, 5B, 5D and 5E). This observation, together with decreased expression of genes related to lipid uptake and lipogenesis under *Bacillus*-treated condition (Fig 5C and 5F), demonstrates that *Bacillus* treatment modulates lipid metabolism in the liver, SAT and MAT, resulting in improved metabolic control through suppression of HFD-induced adiposity in the peripheral tissues.

Imbalance of nutrient status induces gut microbiota dysbiosis, and probiotics exert metabolically beneficial effects by modulating an altered microbiota and its derived metabolites [35]. Many studies have reported that the obesity-associated gut microbiota have an increased efficiency of energy harvest via enhanced colonic fermentation and SCFA production [36,37]. Moreover, SCFAs could be assimilated into host carbohydrates and lipids, being provided as substrates for the production of cholesterol, de novo synthesis of hepatic lipids and gluconeogenesis [38]. In our study, mice fed a HFD showed elevated serum acetate level, which was consistent with previous reports. Although many studies have reported that SCFAs are derived from microbial fermentation of dietary fibers [23], it is also obvious that SCFA levels can be elevated by high fat consumption of host [37], which means that it is ambiguous at present whether, under HFD-fed condition, SCFA levels are affected only by fiber fermentation. We also observed in the present study that elevated serum acetate concentration in HFD-fed mice was reversed by *Bacillus* treatment, which was accompanied by a corresponding downregulation of an acetate receptor, GPR43, in peripheral tissues (S3B and S3C Fig).

We then hypothesized that the decreased levels of serum acetate and GPR43 expression in *Bacillus*-treated mice would reflect the restoration of HFD-induced gut microbiota dysbiosis. It has been demonstrated that the balance between Firmicutes and Bacteroidetes, the dominant phyla in gut microbiota of humans and mice, interrelates with host metabolic status [39]. Many studies have shown that obese human and animal subjects have increased ratio of Firmicutes/Bacteroidetes compared to their lean controls [40–42]. However, a recent study has suggested that the changed Firmicutes/Bacteroidetes ratio in mice has no correlation with high fat content of HFD, and it rather depends on the fiber content of diets [43]. This raises a question whether the Firmicutes/Bacteroidetes ratio is a proper criterion to determine the changes in gut microbiota relating host metabolic status of HFD-fed mice. A few studies on the impact of dietary pattern on gut microbiota alteration have suggested that HFD feeding also results in an increase of *Bacteroides* with corresponding decrease of *Prevotella* [22,44]. In our study, *Bacillus*-treated HFD-fed mice showed no significant change in Firmicutes/Bacteroidetes and *Bacteroides*/*Prevotella* ratios compared to HFD-fed control mice (S3A Fig), from which we could not conclude that *Bacillus* treatment improves HFD-induced dysbiosis. To understand the impact of *Bacillus* treatment on gut microbiota, a more controlled study needs to be conducted, which could minimize the effects exerted by different experimental conditions, for example, sources of diet ingredients or composition of dietary fibers. Moreover, a more comprehensive analysis with extending target bacterial groups should be performed as well.

Together with the fact that probiotics improve metabolic parameters through the modulation of microbiota, it has also been demonstrated that the physiological effects of probiotics on each component of metabolic syndrome are strain-dependent [45]. For instance, three strains of *Lactobacillus reuteri* prevents HFD-induced obesity, insulin resistance and hepatic steatosis in a strain-dependent fashion in Apoe^-/-^ mice [46]. In the present study, we hypothesized that the protective effect of *Bacillus* treatment mentioned above was a combination of the physiological actions exerted by each *Bacillus* strain present in the treated *Bacillus* mixture. Each of five *Bacillus* strains, *B*. *sonorensis* JJY12-3, *B*. *sonorensis* JJY13-1, *B*. *sonorensis* JJY13-3, *B*. *sonorensis* JJY13-8, and *B*. *paralichemiformis* JJY12-8, showed their beneficial effects on metabolic dysfunctions strain-specifically (Fig 6), which indicates that those beneficial effects contribute to the improvement of metabolic dysfunctions by *Bacillus* mixture treatment. We, throughout this study, compared the probiotic effects of VSL#3, a commercial probiotic mixture containing 8 bacterial strains, with those of *Bacillus* mixture. Interestingly, our findings showed that, when treated at the same dose, *Bacillus* mixture appeared to be more potent than VSL#3 in improving metabolic parameters examined in this study.

**Fig 7:**
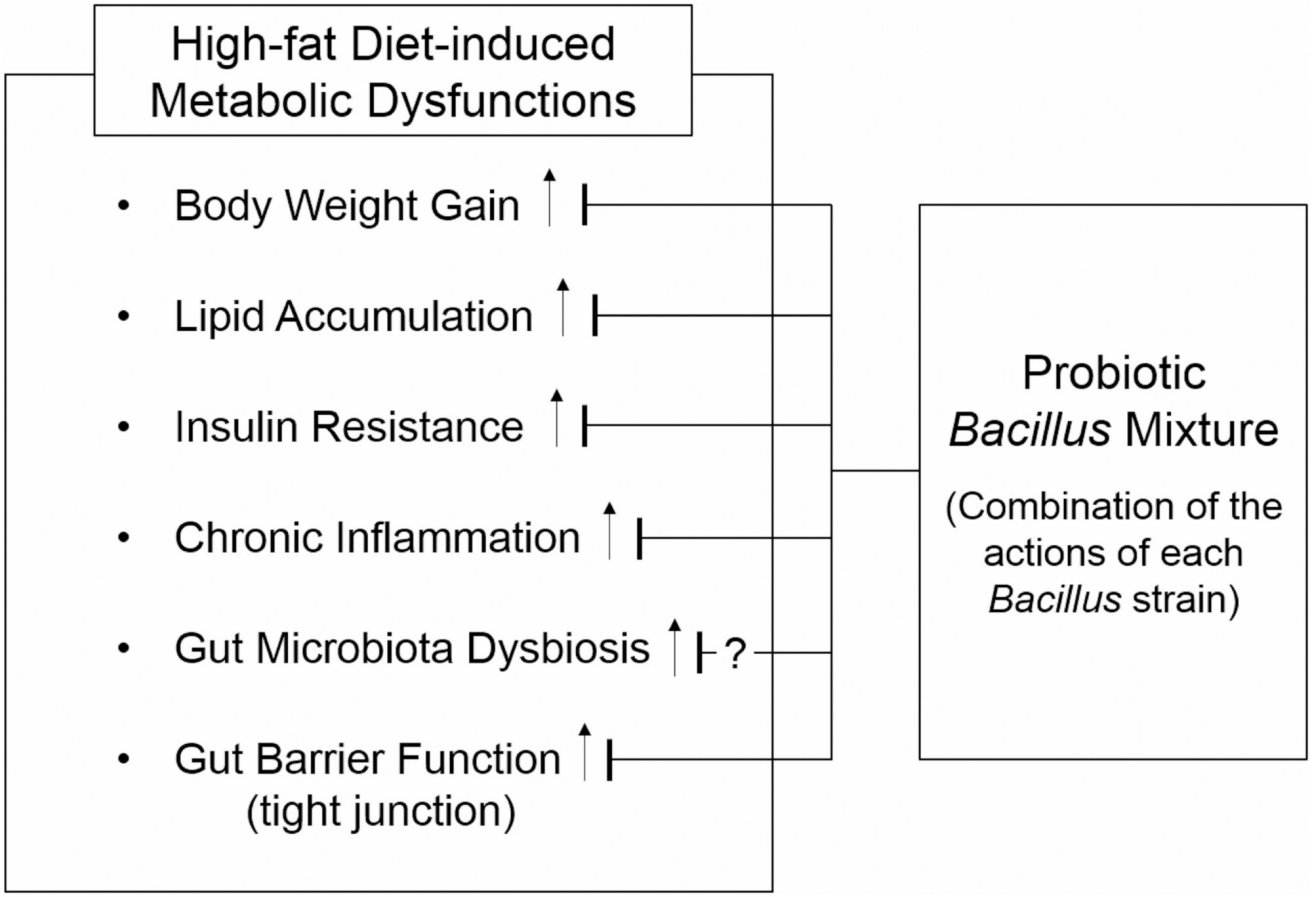
A summary on the protective effects of *Bacillus* probiotics against high-fat diet-induced metabolic disorders.

In summary, our present study demonstrate that *Bacillus* is a promising probiotic candidate which improves HFD-induced obesity, insulin resistance and NAFLD. Our results allow us to propose a model that might explain the *Bacillus*-mediated improvement of metabolic disorders as shown in Fig 7.

As summarized in the diagram, the improving effects of *Bacillus* treatment are contributed by suppression of chronic inflammation, alteration of lipid metabolism, possible improvement of gut barrier function. Our findings conclusively establish probiotic *Bacillus* spp. as potential therapeutic agents for prevention and treatment of metabolic disorders.

## Supporting information

S1 Fig There are no differences in lipogenic gene expression in the liver. Effect of *Bacillus* treatment on hepatic gene expression related to lipid synthesis (n = 5~6). Data present mean ± SD. Differences between experimental groups were analyzed using one-way ANOVA with Tukey’s multiple comparison test. ND: normal chow diet, HF: high-fat diet, PBS: phosphate buffered saline.
(TIF)

S2 Fig There are no differences in lipid oxidative gene expression in subcutaneous and mesenteric adipose tissues. Effect of *Bacillus* treatment on lipid oxidative gene expression in SAT and MAT (n = 5~6). Data present mean ± SD. Differences between experimental groups were analyzed using one-way ANOVA with Tukey’s multiple comparison test. # *p* < 0.05 and ## *p* < 0.01 between ND+PBS and HF+PBS, † *p* < 0.05, †† *p* < 0.01 between HF+PBS and HF+VSL#3, * *p* < 0.05 between HF+PBS and HF+*Bacillus*. ND: normal chow diet, HF: high-fat diet, PBS: phosphate buffered saline, SAT: subcutaneous adipose tissue, MAT: mesenteric adipose tissue.
(TIF)

S3 Fig *Bacillus* treatment might be partially associated with changes of gut microbiota. (A) Changes of fecal microbial composition ratio of Firmicutes/Bacteroidetes and *Bacteroides*/*Prevotella* after 13-week of *Bacillus* treatment (n = 4~5). Changes of levels in (B) serum acetate (n = 3) and (C) GPR43 mRNA expression in the liver, EAT and MAT (n = 5~6). Serum acetate was quantified by gas chromatography. All genes are normalized to expression of Arbp. Data present mean ± SD. Differences between experimental groups were analyzed using one-way ANOVA with Tukey’s multiple comparison test. # *p* < 0.05 and ## *p* < 0.01 between ND+PBS and HF+PBS, † *p* < 0.05, †† *p* < 0.01 between HF+PBS and HF+VSL#3, * *p* < 0.05 and ** *p* < 0.01 between HF+PBS and HF+*Bacillus*. ND: normal chow diet, HF: high-fat diet, PBS: phosphate buffered saline, EAT: epididymal adipose tissue, MAT: mesenteric adipose tissue.
(TIF)

S1 Table Composition of the normal diet and high-fat diet*. (DOCX)

S2 Table Primer sequences for real-time PCR. (DOCX)

S3 Table Product information for western blot. (DOCX)
