# Supplementary figures and images for: Protective effects of Bacillus probiotics against high-fat diet-induced metabolic disorders in mice
Source: PLoS One. 2018 Dec 31;13(12):e0210120. doi: 10.1371/journal.pone.0210120 (PMC6312313; doi:10.1371/journal.pone.0210120)

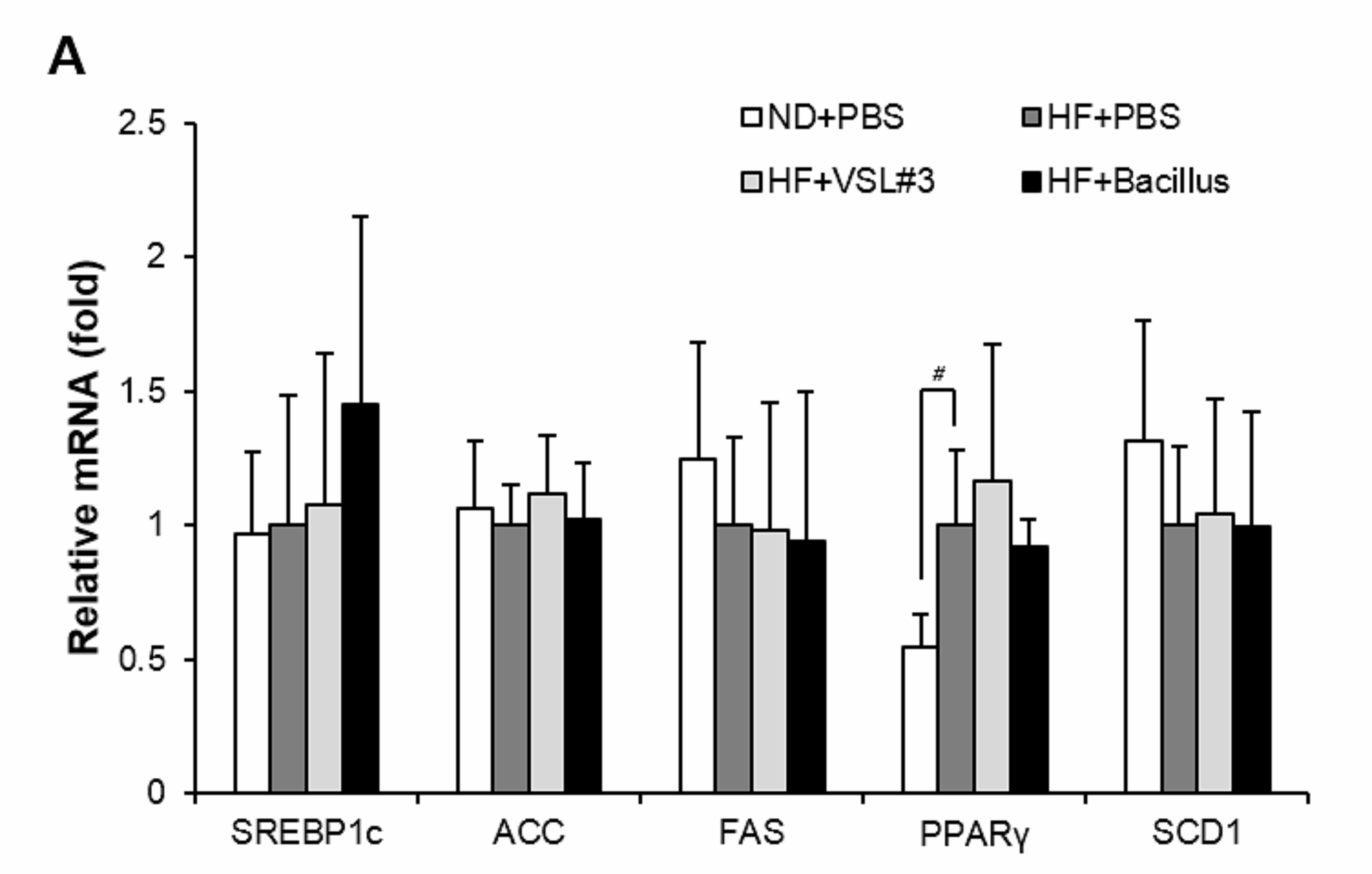

Supplement: S1 Fig — Effect of Bacillus treatment on hepatic gene expression related to lipid synthesis (n = 5~6). Data present mean ± SD. Differences between experimental groups were analyzed using one-way ANOVA with Tukey’s multiple comparison test. ND: normal chow diet, HF: high-fat diet, PBS: phosphate buffered saline. (TIF) [file pone.0210120.s001.tif]

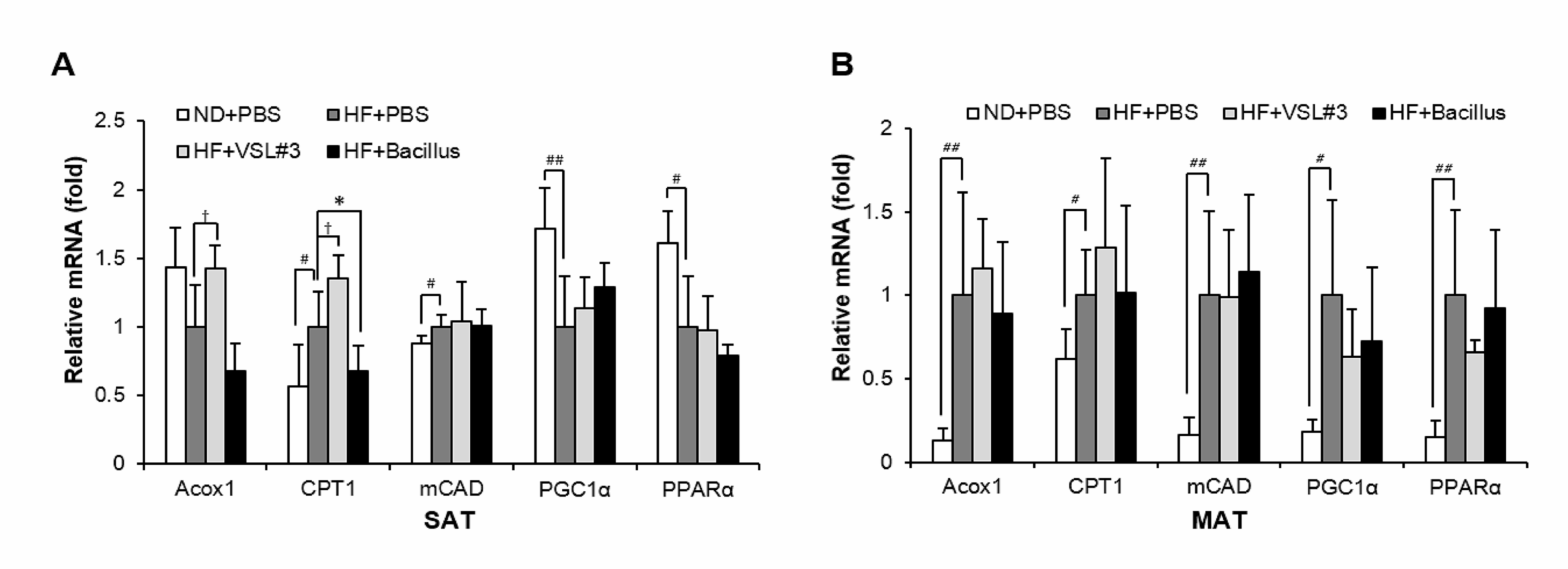

Supplement: S2 Fig — Effect of Bacillus treatment on lipid oxidative gene expression in SAT and MAT (n = 5~6). Data present mean ± SD. Differences between experimental groups were analyzed using one-way ANOVA with Tukey’s multiple comparison test. # p < 0.05 and ## p < 0.01 between ND+PBS and HF+PBS, † p < 0.05, †† p < 0.01 between HF+PBS and HF+VSL#3, * p < 0.05 between HF+PBS and HF+Bacillus. ND: normal chow diet, HF: high-fat diet, PBS: phosphate buffered saline, SAT: subcutaneous adipose tissue, MAT: mesenteric adipose tissue. (TIF) [file pone.0210120.s002.tif]

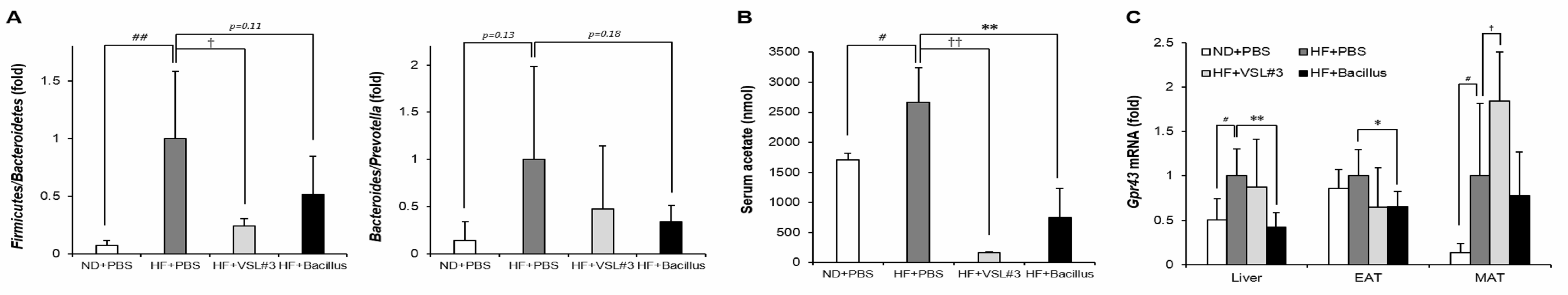

Supplement: S3 Fig — (A) Changes of fecal microbial composition ratio of Firmicutes/Bacteroidetes and Bacteroides/Prevotella after 13-week of Bacillus treatment (n = 4~5). Changes of levels in (B) serum acetate (n = 3) and (C) GPR43 mRNA expression in the liver, EAT and MAT (n = 5~6). Serum acetate was quantified by gas chromatography. All genes are normalized to expression of Arbp. Data present mean ± SD. Differences between experimental groups were analyzed using one-way ANOVA with Tukey’s multiple comparison test. # p < 0.05 and ## p < 0.01 between ND+PBS and HF+PBS, † p < 0.05, †† p < 0.01 between HF+PBS and HF+VSL#3, * p < 0.05 and ** p < 0.01 between HF+PBS and HF+Bacillus. ND: normal chow diet, HF: high-fat diet, PBS: phosphate buffered saline, EAT: epididymal adipose tissue, MAT: mesenteric adipose tissue. (TIF) [file pone.0210120.s003.tif]
