# Supplementary material for: Protective effects of Bacillus probiotics against high-fat diet-induced metabolic disorders in mice
Source: PLoS One. 2018 Dec 31;13(12):e0210120. doi: 10.1371/journal.pone.0210120 (PMC6312313; doi:10.1371/journal.pone.0210120)
Supplement: S1 Table — (DOCX) [file pone.0210120.s004.docx]

| **Normal Diet** | | **High-fat diet** | |
| --- | --- | --- | --- |
| Energy density (kcal/g) | 3.1 | Energy density (kcal/g) | 5.24 |
| Carbohydrate (% w/w) | 44.2 | Carbohydrate (% w/w) | 25.6 |
| Fat (% w/w) | 6.2 | Fat (% w/w) | 34.9 |
| Crude protein (% w/w) | 18.6 | Crude protein (% w/w) | 26.2 |
| Ash (% w/w) | 5.3 | Ash (% w/w) | 5.7 |
| Crude fiber (% w/w) | 3.5 | Dietary fiber (% w/w) | 6.5 |
| Neutral detergent fiber (% w/w) | 14.7 |  |  |

* Diets were not sterilized.
