# Supplementary material for: Protective effects of Bacillus probiotics against high-fat diet-induced metabolic disorders in mice
Source: PLoS One. 2018 Dec 31;13(12):e0210120. doi: 10.1371/journal.pone.0210120 (PMC6312313; doi:10.1371/journal.pone.0210120)
Supplement: S2 Table — (DOCX) [file pone.0210120.s005.docx]

| **Gene** | | **Sequence** |
| --- | --- | --- |
| ACC | Forward | TGACAGACTGATCGCAGAGAAAG |
|  | Reverse | TGGAGAGCCCCACACACA |
| Acox1 | Forward | GTGCAGCTCAGAGTCTGTCCAA |
|  | Reverse | TACTGCTGCGTCTGAAAATCCA |
| CD36 | Forward | TCCTCTGACATTTGCAGGTCTATC |
|  | Reverse | AAAGGCATTGGCTGGAAGAA |
| CPT1 | Forward | CCAATCATCTGGGTGCTGG |
|  | Reverse | AAGAGACCCCGTAGCCATCA |
| FAS | Forward | CTGGACTCGCTCATGGGTG |
|  | Reverse | CATTTCCTGAAGTTTCCGCAG |
| INFγ | Forward | TCAAGTGGCATAGATGTGGAAGAA |
|  | Reverse | TGGCTCTGCAGGATTTTCATG |
| IL-1β | Forward | AGACAGGTCGCTCAGGGTCA |
|  | Reverse | AAGTGGTTGCCCATCAGAGG |
| IL-6 | Forward | TCCAGTTGCCTTCTTGGGAC |
|  | Reverse | AGTCTCCTCTCCGGACTTGT |
| IL-12 | Forward | TCACATCTCATCTCCCCAAA |
|  | Reverse | TCTGCTAACACATTGAGGGG |
| LDLR | Forward | CTGTGATCCGAGTGAGGACG |
|  | Reverse | AGTCTTCTGCTGCAACTCCG |
| MCP-1 | Forward | GTGCTGACCCCAAGAAGGAA |
|  | Reverse | GTGCTGAAGACCTTAGGGCA |
| Occludin | Forward | ATGTCCGGCCGATGCTCTC |
|  | Reverse | TTTGGCTGCTCTTGGGTCTGTAT |
| PPARγ | Forward | AGTGGAGACCGCCCAGG |
|  | Reverse | GCAGCAGGTTGTCTTGGATGT |
| SCD1 | Forward | TCAACTTCACCACGTTCTTCA |
|  | Reverse | CTCCCGTCTCCAGTTCTCTT |
| SREBP1c | Forward | AGCAGCCCCTAGAACAAACAC |
|  | Reverse | CAGCAGTGAGTCTGCCTTGAT |
| TNFα | Forward | CACAAGATGCTGGGACAGTGA |
|  | Reverse | GAGGCTCCAGTGAATTCGGA |
| ZO-1 | Forward | TTTTTGACAGGGGGAGTGG |
|  | Reverse | TGCTGCAGAGGTCAAAGTTCAAG |
| Arbp | Forward | TCACTGTGCCAGCTCAGAAC |
|  | Reverse | AATTTCAATGGTGCCTCTGG |
