# Supplementary material for: Protective effects of Bacillus probiotics against high-fat diet-induced metabolic disorders in mice
Source: PLoS One. 2018 Dec 31;13(12):e0210120. doi: 10.1371/journal.pone.0210120 (PMC6312313; doi:10.1371/journal.pone.0210120)
Supplement: S3 Table — (DOCX) [file pone.0210120.s006.docx]

| **Name** | **Catalog number** |
| --- | --- |
| total Akt | Cell signaling, #9272 |
| phospho (Ser473) Akt | Cell signaling, #4058 |
| total AMPK | Cell signaling, #2532 |
| phospho (Thr172) AMPK | Cell signaling, #2531 |
| adiponectin | Cell signaling, #2789 |
| GAPDH | Bioss, bs-0835R |
| occludin | Bioss, bs-1495R |
| PGC1α | Santa cruz biotechnology, INC., sc-13067 |
| Anti-rabbit IgG, HRP-linked antibody | Cell signaling, #7074 |
